# Integrative analysis of transcriptome and metabolome revealed the mechanisms by which flavonoids and phytohormones regulated the adaptation of alfalfa roots to NaCl stress

**DOI:** 10.3389/fpls.2023.1117868

**Published:** 2023-02-03

**Authors:** Xiaoshan Wang, Juncheng Yin, Jing Wang, Junhao Li

**Affiliations:** Department of Grassland Science, College of Animal Science and Technology, Yangzhou University, Yangzhou, China

**Keywords:** flavonoid, *Medicago sativa*, metabolome, NaCl stress, plant hormones, transcriptome

## Abstract

**Introduction:**

Salinity critically affects the growth and development of alfalfa (Medicago sativa), making it necessary to understand the molecular mechanism of alfalfa’s adaptation to salt stress.

**Methods:**

In this study, alfalfa roots were subjected to salt stress and transcriptomics and metabolomics analyses were performed.

**Results:**

The results showed that flavonoid synthesis, hormone synthesis, and transduction pathways may be involved in the alfalfa salt stress adaptation reaction, and that they are related. Combined analysis of differential genes and differential metabolites found that dihydroquercetin and beta-ring hydroxylase (LUT5), ABA responsive element binding factor 2 (ABF2), protein phosphatase PP2C (PP2C) and abscisic acid (ABA) receptor PYL2 (PYL), luteolinidin was significantly correlated with PP2C and phytochrome-interacting factor 4 (PIF4) and (+)-7-isomethyl jasmonate were significantly correlated with flavonol synthase (FLS) gene. (+)-7-isomethyl jasmonate and homoeriodictyol chalcone were significantly correlated with peroxidase (POD). POD was significantly up-regulated under NaCl stress for 6 and 24 h. Moreover, flavonoids, gibberellin (GA), jasmonic acid (JA) and ABA were suggested to play an important role in alfalfa’s response to salt stress. Further, GA,ABA, and JA may be involved in the regulation of flavonoids to improve alfalfa’s salt tolerance, and JA may be a key signal to promote the synthesis of flavonoids.

**Discussion:**

This study revealed the possible molecular mechanism of alfalfa adaptation to salt stress, and identified a number of salt-tolerance candidate genes from the synthesis and signal transduction pathways of flavonoids and plant hormones, providing new insights into the regulatory network of alfalfa response to salt stress.

## 1 Introduction

Soil salinization is one of the important environmental factors that limit the growth and development of plants. Every year, soil salinization causes about 27.3 billion dollars of economic losses worldwide (Morton et al., 2019). When the ion concentration in the soil is very high, the osmotic potential of the soil is reduced, which makes it difficult for plant roots to absorb water, resulting in osmotic stress. In addition, excessive Na^+^ and Cl^-^ accumulation in plant cells causes ion toxicity and disrupts the normal physiological and biochemical processes of cells (Jia et al., 2019). Since plants grow in soil and cannot escape salt stress, specific salt-tolerance mechanisms, such as osmotic homeostasis, ion balance, and changes in hormone levels and antioxidant enzyme activities, have evolved (Li et al., 2019; Gong, 2021).

Flavonoids are common secondary metabolites in plants. Salt stress can significantly change the transcription level of genes related to flavonoid synthesis, increase flavonoid content, and improve the antioxidant enzyme activity of rice and *Cyclocarya paliurus* (Ithal and Reddy, 2004; Lei et al., 2021). The overexpression of the chalcone synthase (CHS) and flavonol synthase (FLS) genes can increase flavonoid content and enhance salt tolerance in plants (Wang et al., 2018; Wang et al., 2021a). Flavonoids have been found to improve salt resistance in plants by affecting the levels of free radicals and the activity of antioxidant enzymes. The expression of the flavanone hydroxylase (F3H) gene has also been up-regulated after salt stress, promoting the accumulation of flavanols to improve antioxidant enzyme activity in tobacco (Mahajan and Yadav, 2014). The dihydroflavonol reductase (DFR) gene in the anthocyanin branching pathway regulates anthocyanin accumulation and was shown to improve salt tolerance in *Brassica napus* (Kim et al., 2017).

Plant hormones are key signaling molecules in plant responses, in particular, defense responses, to abiotic stress. One study has found that after salt stress, the gibberellin 20 oxidase (GA20ox) gene was down-regulated and the activity of GA20ox oxidase decreased synchronously, resulting in a decrease in gibberellin (GA) content and a decrease in plant growth and development (Zhu et al., 2019). Abscisic acid (ABA) can alleviate the degradation of chlorophyll under salt stress, promote the accumulation of osmotic regulatory substances, and regulate stomata to improve water balance, thereby increasing the salt adaptability of tomato and rice (Shahzad et al., 2017). The ABA responsive element binding factor 2 (ABF2) regulates the stress-induced protein kinase 2 (KIN2) gene in the salt stress transduction pathway to enhance salt resistance in *Arabidopsis* (Zhao et al., 2016). Jasmonic acid (JA) can increase the content of sugars, phenols, and soluble proteins in soybeans and alleviate the negative effects of salt stress (Noor et al., 2022). It has been reported that JA interferes with other plant hormones in stress responses, indicating that JA might be a key signal of the regulatory network of plant hormones (Yang et al., 2019). JA methyl ester (MeJA) inhibits the transcription of B-type RR1, RR11, and RR12, and GA transcription factor PIF3 in the GA transduction pathway. MeJA also promotes the up-regulation of ABA synthesis and the transduction genes 9-cis-epoxycarotenoid dioxygenase (NCED) 4 and NCED 5, as well as the ABA receptors PYL2 (PYL2) and PYL6 (PYL6), and serine/threonine-protein kinase SRK2 (SNRK2). Furthermore, the expression levels of peroxidase (POD), superoxide dismutase (SOD), catalase, and vegetative storage protein 2 (VSP2) genes were increased, improving the salt tolerance of *Nitraria tangutorum* Bobr (Gao et al., 2021).

Transcriptome sequencing is considered an effective method for studying molecular regulatory mechanisms and screening candidate genes for salt resistance in plants (Xu et al., 2021). Metabolomics is the bridge between plant genes and phenotypes. With metabolome detection, changes in plant metabolic profiles can be identified and the response mechanisms of metabolic levels can be analyzed. A number of researchers have used transcriptome analysis methods to identify a large number of salt stress-related genes in plants. In addition, transcription factor families such as MYB, bZIP, and WRKY have been identified to be related to salt tolerance (Zhou et al., 2016; Shi and Gu, 2020; Ouertani et al., 2021; Zhang et al., 2021a; Wang et al., 2022). Metabolomic analysis has revealed that amino acids, fatty acids, sugars, organic acids, plant hormones, and flavonoids play important roles in the salt stress resistance of castor bean, *Haloxylon ammodendron*, soybean, and tobacco (Zhang et al., 2011; Li et al., 2017; Jiao et al., 2018; Panda et al., 2021; Wang et al., 2021b).

Alfalfa (*Medicago sativa* L.) is an important genus of legumes that can both provide nutrients to animals and improve soil fertility (Mokhtari et al., 2017). Alfalfa is considered to be a salt-tolerant plant and is widely cultivated in the coastal areas of eastern China. However, owing to the autotetraploid and cross-pollination characteristics of alfalfa, it is difficult to study the mechanism of alfalfa salt resistance. In recent years, with the development of multi-omics integrative analysis technology and its successful application in plant research, the study of plant salt resistance mechanisms has progressed. In this study, transcriptome and metabolome detection were performed on alfalfa roots after NaCl treatment to study the effects of salt stress on alfalfa gene expression and metabolic pathways. The transcriptome and metabolome data was integrated to explore the specific genes and metabolic pathways that alfalfa adapts under salt stress, providing a new understanding of the regulatory mechanism involved in the response.

## 2 Materials and methods

### 2.1 Plant materials and salt treatment

Alfalfa (WL525) was selected as the experimental plant. Alfalfa seeds were sterilized by soaking in 5% NaClO solution for 3 min, washed four times with distilled water, and then germinated in sand in a dark incubator at 26°C. After three days of incubation, the seedlings were carefully removed from the sand and transplanted into shrinkage holes on foam boards, three seedlings per hole. The foam boards and transplanted seedlings were then transferred to plastic vessels (35 × 28 ×13 cm^3^) with 8.5 L of culture solution. The culture solution contained Ca(NO_3_)_2_ (2.5 mM), KNO_3_ (2.5 mM), MgSO_4_ (1.0 mM), (NH_4_)H_2_PO_4_ (0.5 mM), CuSO_4_ (0.0002 mM), ZnSO_4_ (0.001 mM), EDTA-Fe-Na (0.1 mM), H_3_BO_3_ (0.02 mM), (NH)_4_Mo_27_O_4_ (0.000005 mM), and MnSO_4_ (0.001 mM), had a pH of 7, and was replaced every seven days (Wang and Han, 2007). A 450 μmol m^-2^ s^-1^ bio-sodium lamp provided illumination for 15 h (7:30 am–22:30 pm) every day, and the temperature was kept at 26 or 20°C in light or darkness, respectively. After plants were cultivated for 28 days, and they were divided into two groups. One group was cultured in Hoagland complete nutrient solution as the control group, and the other was supplemented with 150 mmol/L NaCl solution in Hoagland complete nutrient solution. At 0, 1, 6 and 24 h, 1-2 g of alfalfa young roots was collected in 1.7 mL centrifuge tubes. After collection, the samples were placed in liquid nitrogen. Then, the samples were divided into two parts. One part was stored in a -80°C refrigerator for subsequent transcriptome sequencing and metabolome detection, and the other part was used to RT-qPCR verification.

### 2.2 Measurement of physiological and biochemical indicators

Peroxidase (POD) activity was determined by guaiacol colorimetry; superoxide dismutase (SOD) activity was determined by the nitrogen blue tetrazole colorimetric method; catalase (CAT) activity was determined by the ammonium molybdate colorimetric method; malonaldehyde (MDA) was determined by the thiobarbiturate method; proline (PRO) was determined by ninhydrin colorimetry, and soluble protein by the bicinchoninic acid (BCA) method (Cai, 2012). The above indices were determined using the COMIN kit (COMIN, Suzhou, China).

### 2.3 Transcriptome sequencing

Total RNA was extracted from 12 root samples using a TreliefTM RNAprep Pure Plant Kit (Tsingke, Nanjing, China), and a transcriptome library was constructed. The quality and concentration of RNA were determined using 1% agarose gel electrophoresis, Agilent2100 (BGI, Shenzhen, China), and NanoDrop (BGI, Shenzhen, China). Sequencing was performed on the DNBSEQ platform (BGI, Shenzhen, China), using the latest published genome data from Zhongmu No.1 (https://figshare.com/articles/dataset/Medicago_sativa_genome_and_annotation_files/12623960) as a reference, and using SOAPnuke (BGI, Shenzhen, China) and Trimmomatic (BGI, Shenzhen, China) for statistical filtering. The resulting high-quality reads (clean reads), and datasets can be found in online repositories (https://www.ncbi.nlm.nih.gov/sra/,PRJNA892760, **Table S1**). The detection of differentially expressed genes (DEGs) was performed according to the Kyoto Encyclopedia of Genes and Genomes (KEGG) and Gene Ontology (GO). The Phyper function in R software was used for enrichment analysis, the P-value was calculated, and then the P-value was corrected by false discovery rate. Generally, a Q-value ≤ 0.05 was regarded as significant enrichment.

### 2.4 Metabolomic analysis

Metabolites in the alfalfa roots were extracted and analyzed using liquid chromatography (2777CUPLC system; BGI, Shenzhen, China) and mass spectrometry (Xevo G2-XS QTOF; BGI, Shenzhen, China). To obtain reliable data, quality control (QC) samples were added to the detection process. The original mass spectrometry data were imported into the Progenesis QI software (Version 2.2; BGI, Shenzhen, China) for peak extraction. The KNN method was used to fill the missing values and remove low-quality ions (removing the ions missing in more than 50% QC samples or in more than 80% actual samples). Finally, univariate and multivariate analyses were performed using the metabolomic data analysis software Metax (BGI, Shenzhen, China). The multivariate analysis was used to analyze the variable important for the projection of the first two principal components of the PLS-DA model. The univariate analysis was combined with fold-change and Q values to screen metabolites with differential expression (VIP ≥ 1; fold change ≥ 1.2 or ≤ 0.8333; Q-value < 0.05). The metabolites at the intersection of the three were selected as the final differential accumulation metabolites (DAMs), and the thus-obtained inter-group differential metabolites were annotated by KEGG.

### 2.5 Transcriptome and metabolome correlation network analysis

A Spearman correlation analysis was performed on the transcriptome and metabolome, and the genes and metabolites with absolute correlation coefficient > 0.99 and p-value < 0.01 were screened. Cytoscape was used to construct the network map.

### 2.6 Real-time quantitative PCR

FastKing gDNA Dispelling RT SuperMix (TIANGEN, Beijing, China) was used to transcribe the RNA to cDNA. Primer Premier 5.0 was used with MSActin as an internal reference. RT-qPCR was performed using the 2 × TSINGKE Master qPCR Mix (SYBR Green I; Tsingke, Nanjing, China) on a MiniOpticon TM Real-Time PCR System (Bio-Rad, Hercules, CA, USA). Each biological replicate consisted of three technical replicates, and relative expression was calculated according to the 2^-ΔΔCT^ method.

## 3 Results

### 3.1 Transcriptome sequencing for alfalfa roots under short-term NaCl stress

**Figure 1:**
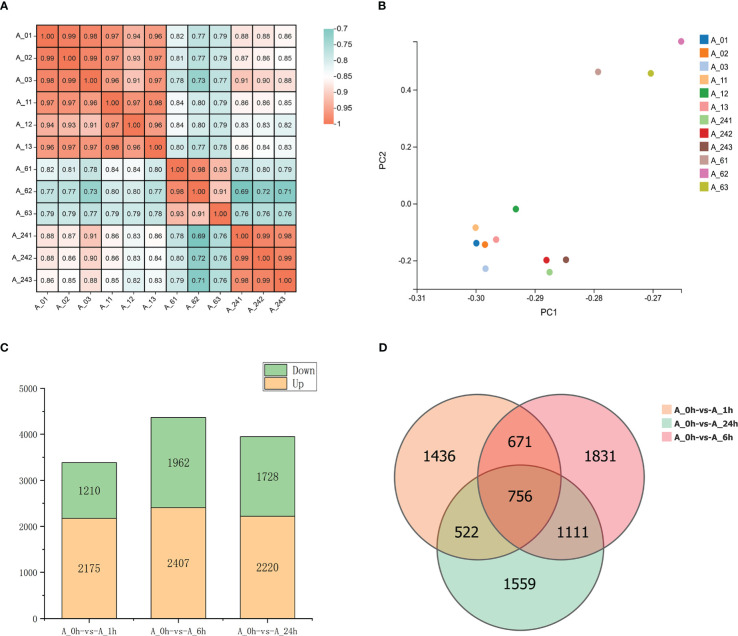
**(A)** Heat map showing the correlation between samples. **(B)** PCA results of transcriptome of each treatment. **(C)** The number of DEGs upregulated and downregulated in different treatments. **(D)** Venn diagram of DEGs. The four time treatments of 0, 1, 6 and 24h were denoted as A_0h, A_1h, A_6h and A_24h respectively.

The roots of alfalfa were collected for transcriptome sequencing after treatment with 150 mM NaCl solution for 0, 1, 6, and 24 h, and 77.04 Gb of clean reads were obtained. The sequencing results of the samples treated with NaCl for different periods were highly correlated (r^2^ > 0.91) and repeatable (**Figure 1A**). Principal component analysis (PCA) results revealed that the PCA values at 6 h under NaCl stress were significantly different from those at 0 and 1 h. Moreover, PCA results were separated in the direction of PC1 at 24 h (**Figure 1B**), which was consistent with the correlated heat map results (**Figure 1A**). The results indicated a significant difference in the transcription levels at 6 and 24 h of NaCl treatment compared to those at 0 and 1 h of treatment. Compared to the NaCl treatment at 0 h, 3385, 4369, and 3948 DEGs were detected at 1, 6, and 24 h of treatment, respectively. At 1 h of NaCl treatment, 2175 DEGs were up-regulated and 1210 DEGs were down-regulated. A total of 2407 DEGs were up-regulated and 1962 DEGs were down-regulated at 6 h of treatment. At 24 h, 2220 DEGs were up-regulated and 1728 DEGs were down-regulated. There were 756 common DEGs under NaCl treatment at 1, 6, and 24 h, among which 352 remained up-regulated and 300 remained down-regulated (**Figures 1C, D**).

GO enrichment analysis revealed that the DEGs were mainly enriched in signal transduction, stimulation response, regulation of cellular processes, protein kinase activity, defense response, naringin CHS activity, cell stimulation response, flavonoid biosynthesis, and other functional units under NaCl stress for 1 h (**Figure S1**). Under NaCl stress for 6 h, DEGs were mainly enriched in functional units such as oxidoreductase activity, reactive oxygen species metabolism process, oxidative stress response, signal transduction, flavonoid biosynthesis process, JA biosynthesis process, hormone-mediated signaling pathway, and ABA-activated signaling pathway (**Figure S1**). Under NaCl stress for 24 h, DEGs were mainly enriched in oxidoreductase activity, dioxygenase activity, reactive oxygen species metabolism process, hydrogen peroxide catabolic process, POD activity, antioxidant activity, ABA binding, and hormone-binding functional units (**Figure S1**). The 756 common DEGs under NaCl stress at 1, 6, and 24 h were mainly enriched in functional units such as oxidoreductase activity, hormone-mediated signaling pathway, ABA binding, protein dephosphorylation regulation, cell response to hormone stimulation, ABA response, hormone binding, and cell response to endogenous stimulus (**Figure S1**). By comparing the results of DEGs and analyzing the GO enrichment results of common DEGs at 1, 6, and 24 h of NaCl stress, we found that functional units such as the oxidoreductase regulation process, flavonoid synthesis of secondary metabolites, as well as hormone synthesis and transduction, are important pathways for alfalfa adaptation to NaCl stress.

### 3.2 Analysis of metabolite detection under short-term NaCl stress

**Figure 2:**
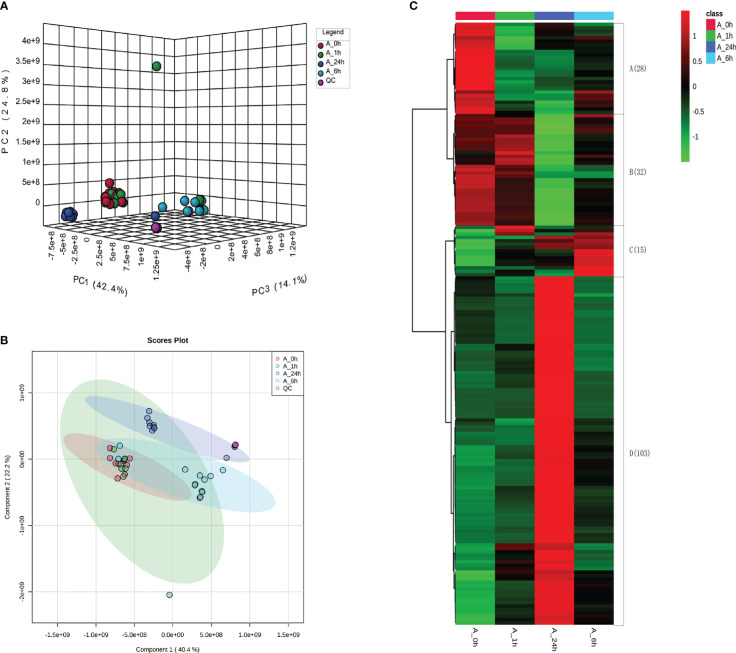
**(A)** Principal component analysis (PCA) results of metabolome of each treatment. **(B)** The results of metabolome partial least squares (PLS-DA) analysis. Partial least squares (PLS-DA) model was constructed with MetaX software for the analysis of the results. The model parameters R2 and Q2 had high values (**Table S2**). The 200 displacement tests of the model parameters R2 and Q2 showed that the current PLS-DA model was reliable. **(C)** Heat map analysis of DAMs in different treatments.

After treatment with 150 mM NaCl for 0, 1, 6, and 24 h, the root tips of alfalfa were collected for untargeted metabolite detection using liquid chromatography-mass spectrometry. PCA results showed that the QC samples clustered to one point, indicating that the instrument was stable, and the collected data were of high quality (**Figure 2A**). The three principal components PC1, PC2, and PC3 accounted for 42.4, 24.8, and 14.1% of the total variation, respectively (**Figure 2A**). The PCA values of alfalfa root tips under NaCl stress at 0 and 1 h were closely clustered, whereas the PCA values of alfalfa root tips under NaCl stress at 6 and 24 h were separated in the direction of PC1 and PC3 (**Figure 2A**). The results showed that the metabolite levels at 6 and 24 h of NaCl stress were significantly different from those at 0 and 1 h, and the metabolite levels at 0 h and 1 h of NaCl stress were similar. The PLS-DA results showed that the components at 6 and 24 h were separated from the components at 0 and 1 h of NaCl stress, which is consistent with the PCA results (**Figure 2B**).

In the present study, 32 DAMs were found under NaCl stress for 1 h, of which 25 were down-regulated and 7 were up-regulated. Twenty-nine DAMs were found under NaCl stress for 6 h, of which 11 were down-regulated and 18 were up-regulated. A total of 153 DAMs were found under NaCl stress for 24 h, of which 50 were down-regulated and 103 were up-regulated. The results showed that differential metabolites increased significantly with increasing NaCl stress duration (**Figure S2**). A total of 178 common metabolites were detected from the DAMs of alfalfa roots treated by NaCl for 1, 6, and 24 h, which were divided into four categories according to their expression changes. The expression levels of 28 metabolites decreased under NaCl stress for 1 h, 6 h, and 24 h. The expression levels of 32 metabolites remained unchanged at 1 and 6 h under NaCl stress but decreased significantly at 24 h. The expression levels of 15 metabolites increased first and then decreased during 1–24 h of NaCl stress. The expression levels of 103 metabolites remained unchanged at 1 and 6 h under NaCl stress, but significantly increased at 24 h (**Figure 2C**; **Tables S3, S4**).

### 3.3 Integrative analysis of transcriptome and metabolome for alfalfa roots under short-term NaCl stress

**Figure 3:**
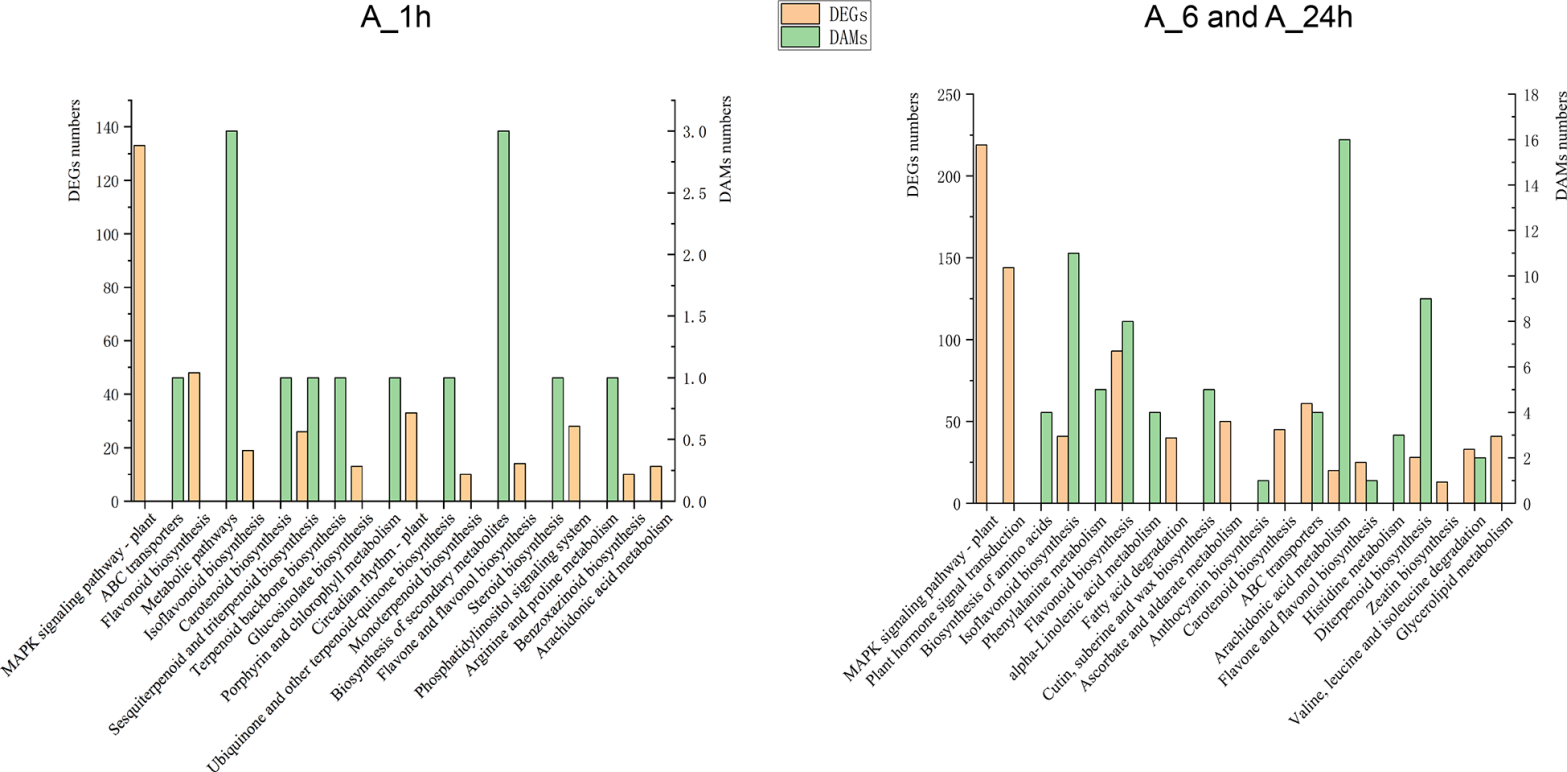
KEGG coenrichment analysis of DEGs and DAMs treated with NaCl stress at different times (Q-value < 0.05). According to the changes of transcription and metabolism levels of the samples, alfalfa had no obvious response to salt stress at 1h, so 1h and the following 6 and 24h were analyzed separately. A_6h and A_24h were the two time points of KEGG enrichment pathway, which were combined for metabolome and transcript co-enrichment analysis.

To determine the correlation between DEGs and DAMs under NaCl stress, KEGG pathway analysis was used to conduct a correlation analysis of the transcriptome and metabolome detection data to elucidate a common enrichment pathway. KEGG enrichment pathways showed one common enrichment pathway in DEGs and DAMs under NaCl stress at 1 h. There were seven common enrichment pathways at 6 and 24 h of NaCl stress (**Figure 3**). The data analyses of transcriptome and metabolome also showed that the expression levels of genes and metabolites were similar at 0 and 1 h under NaCl stress, whereas at 6 and 24 h, these were significantly different from those at 0 h and 1 h (**Figures 1A, B**, **2A, B**). Moreover, the contents of MDA, osmotic substances PRO and soluble protein, and the activities of antioxidant enzymes POD, SOD, and CAT were also measured at 0, 1, 6, and 24 h of NaCl stress. The contents of MDA, PRO, and soluble protein and the activities of POD, SOD, and CAT did not change significantly at 1 h of NaCl stress compared with those at 0 h. The content of MDA and the activities of POD and SOD increased significantly at 6 and 24 h of NaCl stress (**Figure S3** and **Table S5**). The results showed that alfalfa plants had no obvious response to NaCl stress before 1 h, but a clear response after 1 h. At 6 and 24 h of NaCl stress, common pathways of DEGs and DAMs were isoflavone biosynthesis, flavonoid biosynthesis, ABC transporter, arachidonic acid metabolism, flavonoid and flavonol biosynthesis, and diterpenoid biosynthesis, as well as degradation of valine, leucine, and isoleucine. In addition, DEGs were also enriched in the MAPK signaling pathway, plant hormone signal transduction, carotenoid biosynthesis, and zeatin biosynthesis. DAMs were also enriched in alpha-linolenic acid metabolism and anthocyanin biosynthesis (**Figure 3**).

### 3.4 Effects of short-term NaCl stress on flavonoid biosynthetic pathways

**Figure 4:**
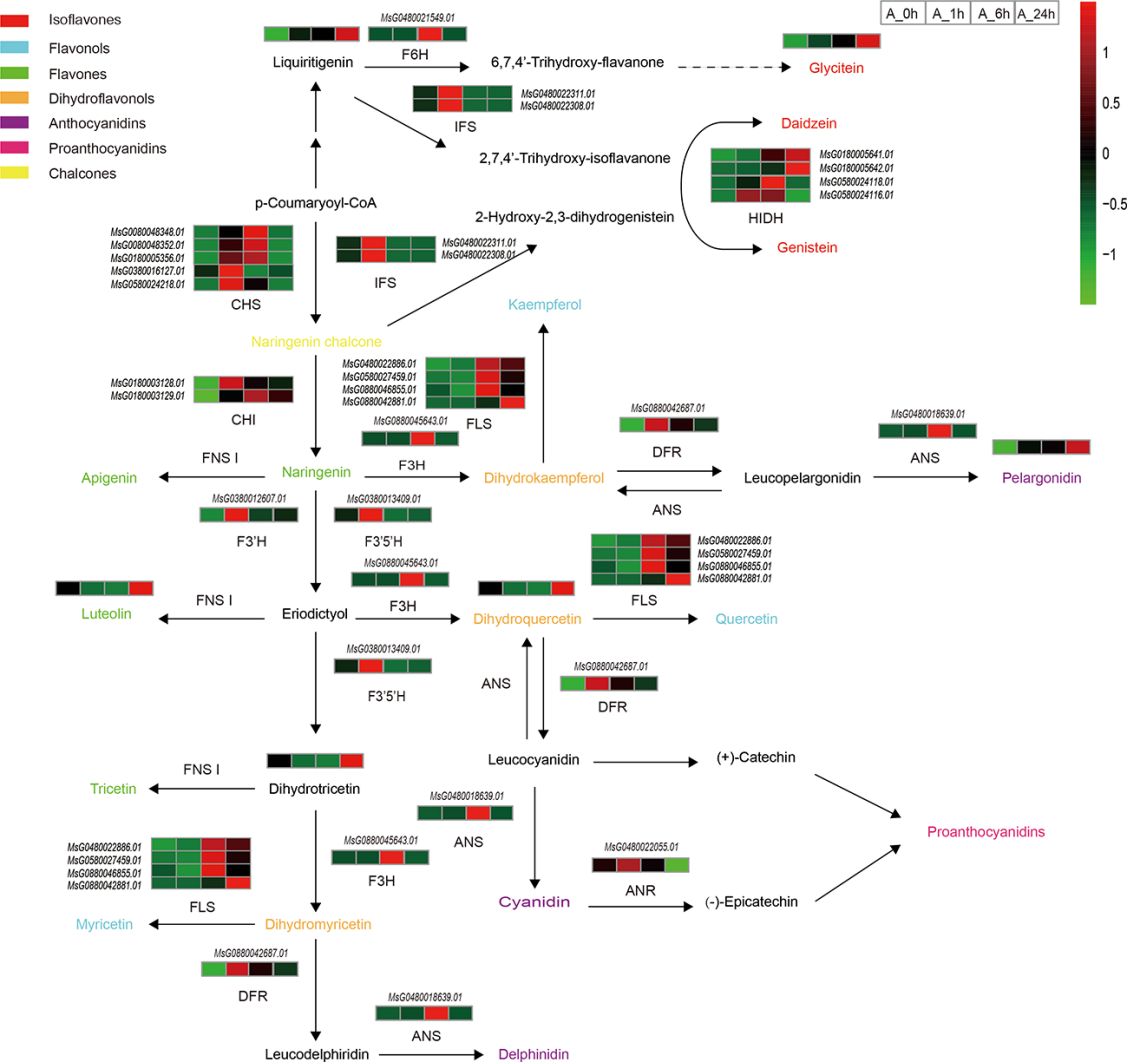
Changes of important genes and metabolites in flavonoid biosynthesis under NaCl stress. Path network diagram were mapped based on KEGG and related literature. Italics indicate gene ids that label enzymes or transcription factors, the rest are unlabeled metabolites, and different colors indicate different types of flavonoids.

Flavonoids are common secondary metabolites in plants and are closely related to plant stress tolerance. Under NaCl stress, the major metabolites, and regulatory genes involved in the flavonoid biosynthesis pathway of alfalfa were significantly altered (**Figure 4**; **Tables S6, S7**). With the duration of NaCl stress, the expression levels of most enzymes involved in flavonoid synthesis first increased and then decreased (**Figure 4**). Accumulation of most metabolites reached its highest level at 24 h of NaCl stress (**Figure 4**). This is because in the early stage of NaCl stress, the enzymes genes related to flavonoid synthesis were up-regulated, which led to the rapid initial increase of metabolite synthesis (**Figure 4**). With continuous NaCl stress, plants gradually adapted, and the expression of related enzymes genes decreased, but the accumulation of metabolites still increased. Chalcone synthase (CHS) is a key rate-limiting enzyme in the flavonoid biosynthesis pathway. In the present study, the expression levels of five genes (MsG0080048348.01, MsG0080048352.01, MsG0180005356.01, MsG0380016127.01, and MsG0580024218.01) encoding CHS were significantly up-regulated at 1 or 6 h of NaCl stress. Two genes (MsG0180003128.01 and MsG0180003129.01) encoding chalconeisomerase (CHI) always remained up-regulated during NaCl stress. The expression level of the flavanone 3-hydroxylase (F3H, MsG0880045643.01) gene was the highest under 6 h NaCl stress (**Figure 4**). The accumulation of dihydroquercetin, an intermediate product of flavonoid biosynthesis, first decreased and then increased with the duration of NaCl stress, and reached its maximum value at 24 h (**Figure 4**). The flavonol synthetase pathway is a branch of the flavonol synthesis pathway. In the present study, four genes (MsG0480022886.01, MsG0580027459.01, MsG0880042881.01, and MsG0880046855.01) encoding flavonol synthetase (FLS) were up-regulated at 6 and 24 h under NaCl stress (**Figure 4**). In the flavone branch pathway, the accumulation of luteolin first decreased and then increased with the duration of NaCl stress, and accumulation reached its highest value at 24 h (**Figure 4**). Under NaCl stress, the expression of dihydroflavonol reductase (DFR, MsG0880042687.01) was up-regulated in the anthocyanin synthesis pathway branch, reaching its highest level at 1 h. Interestingly, the expression of anthocyanin synthase (ANS, MsG0480018639.01) was only up-regulated at 6 h. In the flavonoid synthesis pathway, the accumulation of the metabolite pelargonidin, the intermediate product of flavonoid synthesis, gradually increased with the duration of NaCl stress, and reached its maximum value at 24 h. The anthocyanidin reductase (ANR, MsG0480022055.01) gene of the proanthocyanidin synthesis pathway, another branch pathway of flavonoid synthesis, was up-regulated at 1 h and down-regulated at 24 h of NaCl stress. (**Figure 4**). The isoflavone synthesis pathway is yet another branch pathway of flavonoid synthesis. The accumulation of liquiritigenin and glycitein in this pathway gradually increased with the duration of NaCl stress (**Figure 4**).

### 3.5 Effects of short-term NaCl stress on phytohormone synthesis and transactivation

**Figure 5:**
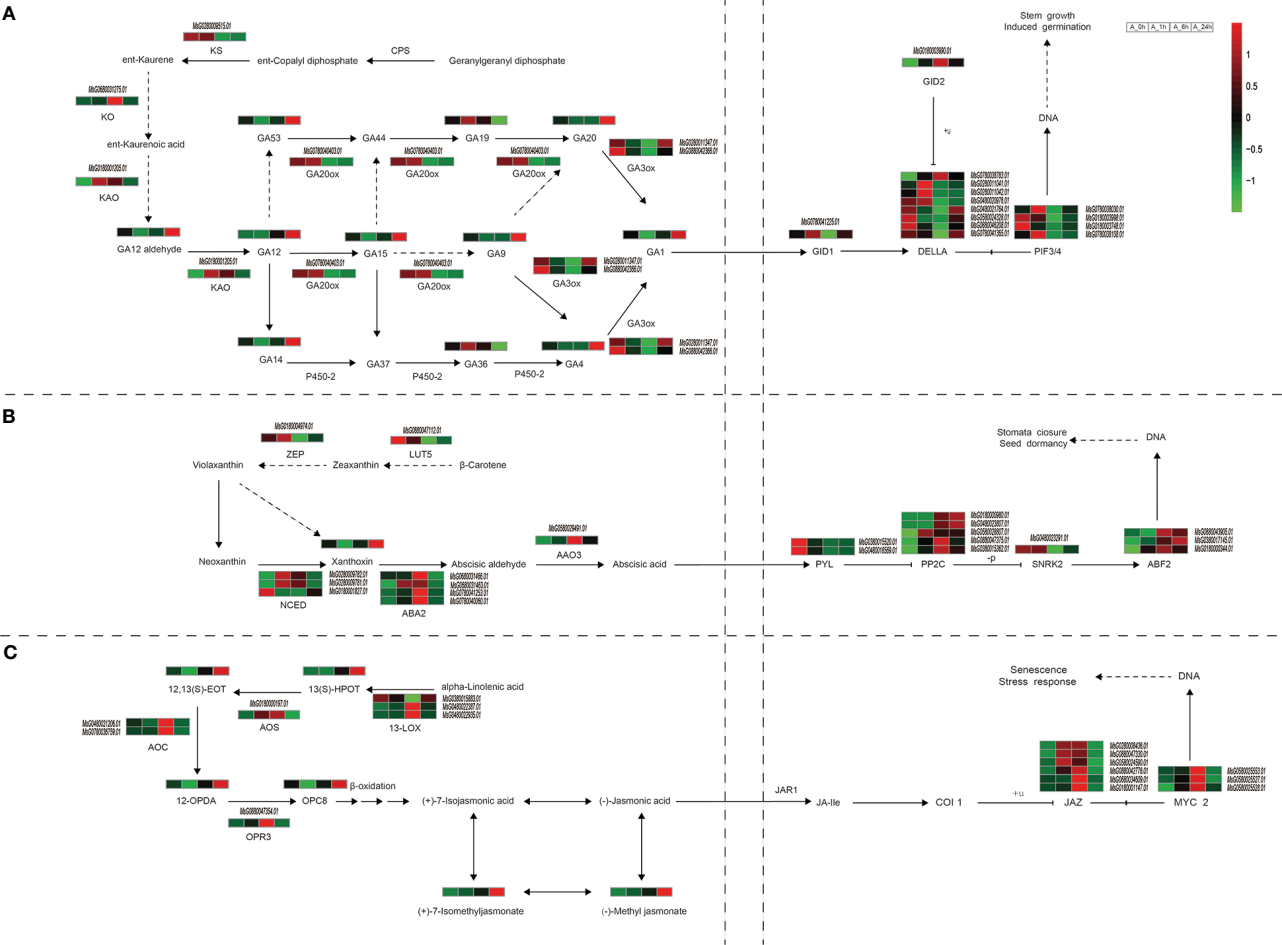
Changes of important genes and metabolites in plant hormone synthesis and transduction under NaCl stress. **(A)** GA synthesis and transduction process. **(B)** ABA synthesis and transduction process. **(C)** JA synthesis and transduction process. Pathways were mapped based on KEGG and related literature. Italics indicate gene ids that label enzymes or transcription factors, the rest are unlabeled metabolites.

Phytohormones are the integrated centers of plant response to environmental stress, and the complex signal network formed by different hormones can achieve fine regulation of stress response. In the present study, the expression levels of genes related to the synthesis and transduction pathways of key hormones GA, ABA and JA, and the accumulation of intermediate metabolites during their synthesis were altered in alfalfa under NaCl stress (**Tables S8, 9**). The gibberellin-20 oxidase (GA20ox, MsG0780040403.01) gene was down-regulated in the GA synthesis pathway for 6 and 24 h. The accumulation of gibberellin 12 (GA 12), gibberellin 1 (GA 1), gibberellin 4 (GA 4), gibberellin 9 (GA 9), gibberellin 14 (GA 14), gibberellin 15 (GA 15), gibberellin 20 (GA 20), and gibberellin 53 (GA 53) showed a trend of first decreasing and then increasing during NaCl stress, with the highest levels reaching at 24 h (**Figure 5A**). This result may be related to the up-regulation of ent-kaurene oxidase (KO, MsG0680031275.01) and ent-kaurenoic acid monooxygenase (KAO, MsG0180001205.01) genes at 1 and 6 h of NaCl stress (**Figure 5A**). In the present study, the expression of gibberellin 3beta-dioxygenase (GA3ox, MsG0280011347.01 and MsG0880042366.01) gene first decreased and then increased. After 1 h of NaCl stress, the levels of GA19 and GA36 were significantly decreased, while the levels of GA9, GA1 and GA4 were significantly increased, indicating that active GA1 and GA4 may be synthesized mainly through GA9 after 1 h NaCl stress. In our study, seven of the eight DELLA protein genes (MsG0280011041.01, MsG0280011042.01, MsG0480020976.01, MsG0480021764.01, MsG0580024328.01, MsG0780041365.01, and MsG0880046208.01), four PIF3/4 genes, and one GID1 gene (MsG0780041225.01) were down-regulated at 6 h of NaCl, while the expression levels of most DELLA genes at 24 h were significantly higher than at 0 and 6 h (**Figure 5A**).

Three genes (MsG0280009782.01, MsG0280009781.01, and MsG0180001827.01) encoding 9-cis-epoxycarotenoid dioxygenase (NCED) in the ABA synthesis pathway of alfalfa were significantly up-regulated under NaCl stress for 1 h and 6 h (**Figure 5B**), but the ABA content did not change significantly (**Figure 5B**). Both PYL (MsG0380015520.01 and MsG0480018569.01) and SnRK2 (MsG0480023291.01) genes were down-regulated under NaCl stress for 6 and 24 h, but all genes encoding PP2C (MsG0180000980.01, MsG0380015362.01, MsG0480023807.01, MsG0580028807.01, and MsG0880047375.01) and ABF2 (MsG0380017145.01, MsG0880043905.01, and MsG0180000344.01) were up-regulated (**Figure 5B**). These results indicate that salt stress could affect the gene expression of some enzymes and the accumulation of some intermediate metabolites in the ABA synthesis pathway, but has no significant effect on the content of ABA in roots.

In the JA synthesis pathway, two of the three genes encoding 13s-lipoxygenase (13-Lox) (MsG0480022387.01 and MsG0480022935.01), and two genes (MsG0780036759.01 and MsG0480021206.01) encoding allene oxide cyclase (AOC) were up-regulated under NaCl stress for 6 h. Allene oxide synthase (AOS, MsG0180000197.01), and 12-oxophytodienoic acid reductase (OPR3, MsG0880047354.01) in the JA synthesis pathway were up-regulated under NaCl stress for 1 h and 6 h (**Figure 5C**). This may lead to the accumulation of synthetic intermediates such as 12, 13(S)-EOT, 12-OPDA, and OPC8; these were down-regulated at 1 h compared with those at 0 h, and decreased first and then increased during 24 h NaCl stress, at which time their concentrations were the highest (**Figure 5C**). The metabolites 13(S)-HPOT, methyl jasmonate, and (+)-7-Isomethyljasmonate showed a gradual increase in accumulation within 24 h of NaCl stress. All of the six genes (MsG0180001147.01, MsG0280008436.01, MsG0580024590.01, MsG0680034609.01, MsG0880042776.01, and MsG0880047330.01) encoding Jasmonate-ZIM domain genes (JAZ) were up-regulated at 6 h NaCl stress, and four of these (MsG0280008436.01, MsG0580024590.01, MsG0880042776.01, and MsG0880047330.01) were up-regulated at 1 h NaCl stress. All the genes (MsG0580025527.01, MsG0580025528.01, and MsG0580025553.01) encoding MYC2 were up-regulated at 1 and 6 h NaCl stress (**Figure 5C**). The results showed that the expression levels of some enzyme and transduction genes in the JA synthesis pathway were significantly up-regulated by NaCl stress at 6 h, and the accumulation of the intermediates methyl jasmonate and 7-isomethyl jasmonate significantly increased.

### 3.6 Gene and metabolite related networks

**Figure 6:**
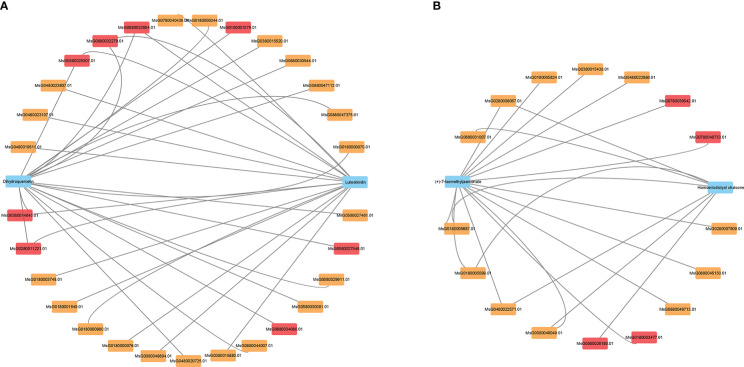
DEGs and DAMs association analysis network. **(A, B)** Correlation analysis of DEGs and DAMs at 6 and 24 h NaCl stress. Blue represents metabolites, red (p < 0.01) and yellow (p < 0.05) are genes.

Correlation analysis of DEGs and DAMs on the major enrichment pathways in alfalfa at 6 and 24 h of NaCl stress resulted in the construction of a gene–metabolite association network related to flavonoids and hormones (**Table S10**). Dihydroquercetin in the flavonoid synthesis pathway; ABF2 (MsG0180000344.01), PP2C (MsG0880047375.01) and PYL (MsG0380015520.01) genes in the ABA signal transduction pathway; and the β-cyclic hydroxylase (LUT5, MsG0880047112.01) gene in the gibberellin synthesis pathway were significantly correlated (**Figure 6A**). The flavonoid luteolinidin was significantly correlated with the genes PIF4 (MsG0180003748.01) and PP2C (MsG0180000980.01, MsG0480023807.01) in the gibberellin signal transduction pathway. Homoeriodictyol chalcone in flavonoid synthesis pathway and (+)-7-isomethyl jasmonate in JA synthesis pathway coexisted with POD (MsG0280008067.01) and pathogenesis-related protein 1 (PR1, MsG0480022571.01) (**Figure 6B**). In addition, (+)-7-isomethyl jasmonate was also associated with FLS (MsG0480022886.01), JA downstream vegetative storage protein 2 (VSP2, MsG0380015438.01) gene, cis zeatin synthesis dimethyl allyl transferase (miaA, MsG0780039542.01) gene, and ethylene responsive transcription factor 1 (ERF1, MsG0780040733.01, **Figure 6B**). These results suggested that flavonoid synthesis is highly correlated with DEGs and the metabolites of phytohormone syntheses, such as GAs, JA, and ABA. Alfalfa could be resistant to salt stress through the interaction between flavonoids and phytohormones, which may play a key role in the process of salt stress tolerance in plants.

### 3.7 RT-qPCR validation

**Figure 7:**
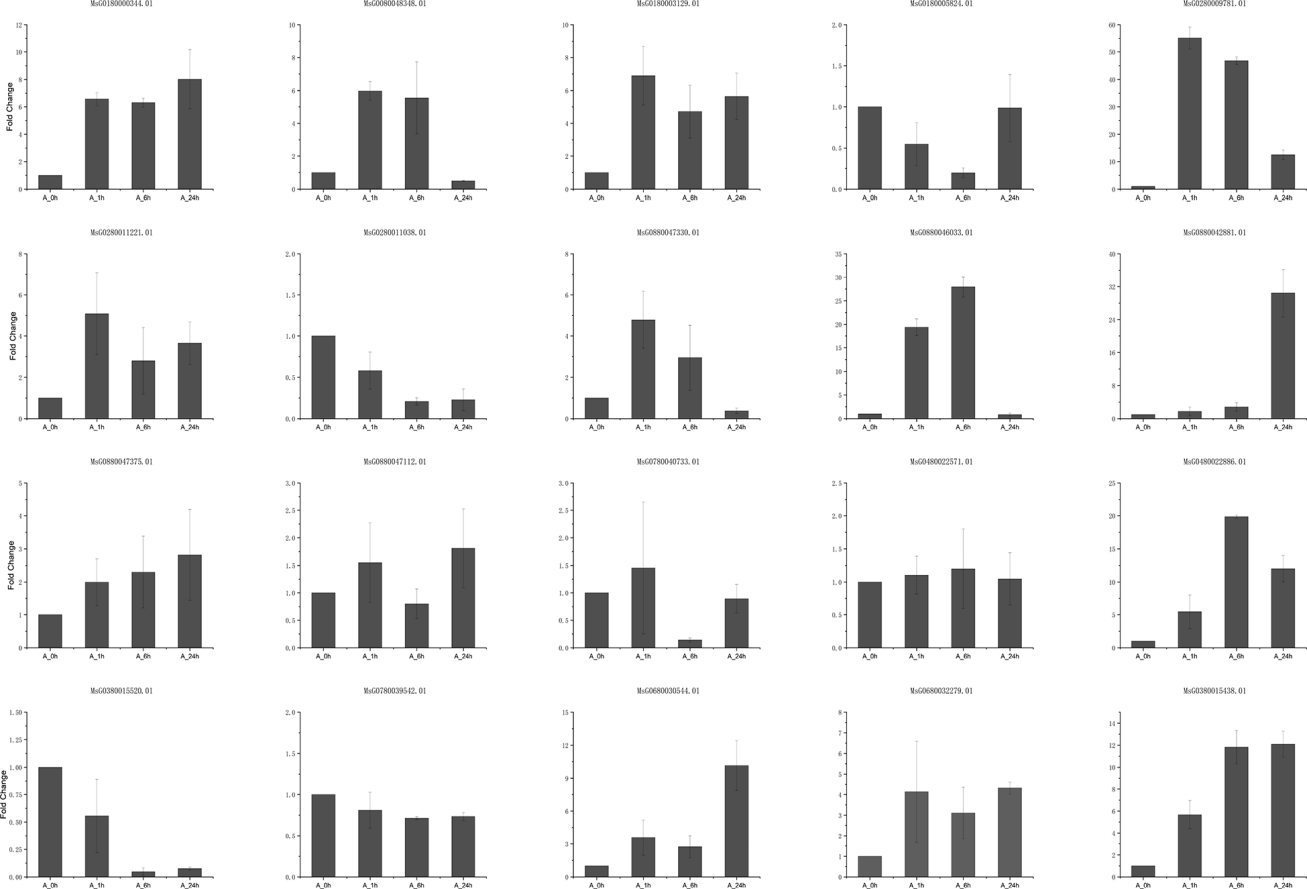
The results of RT-qPCR with twenty genes.

**Figure 8:**
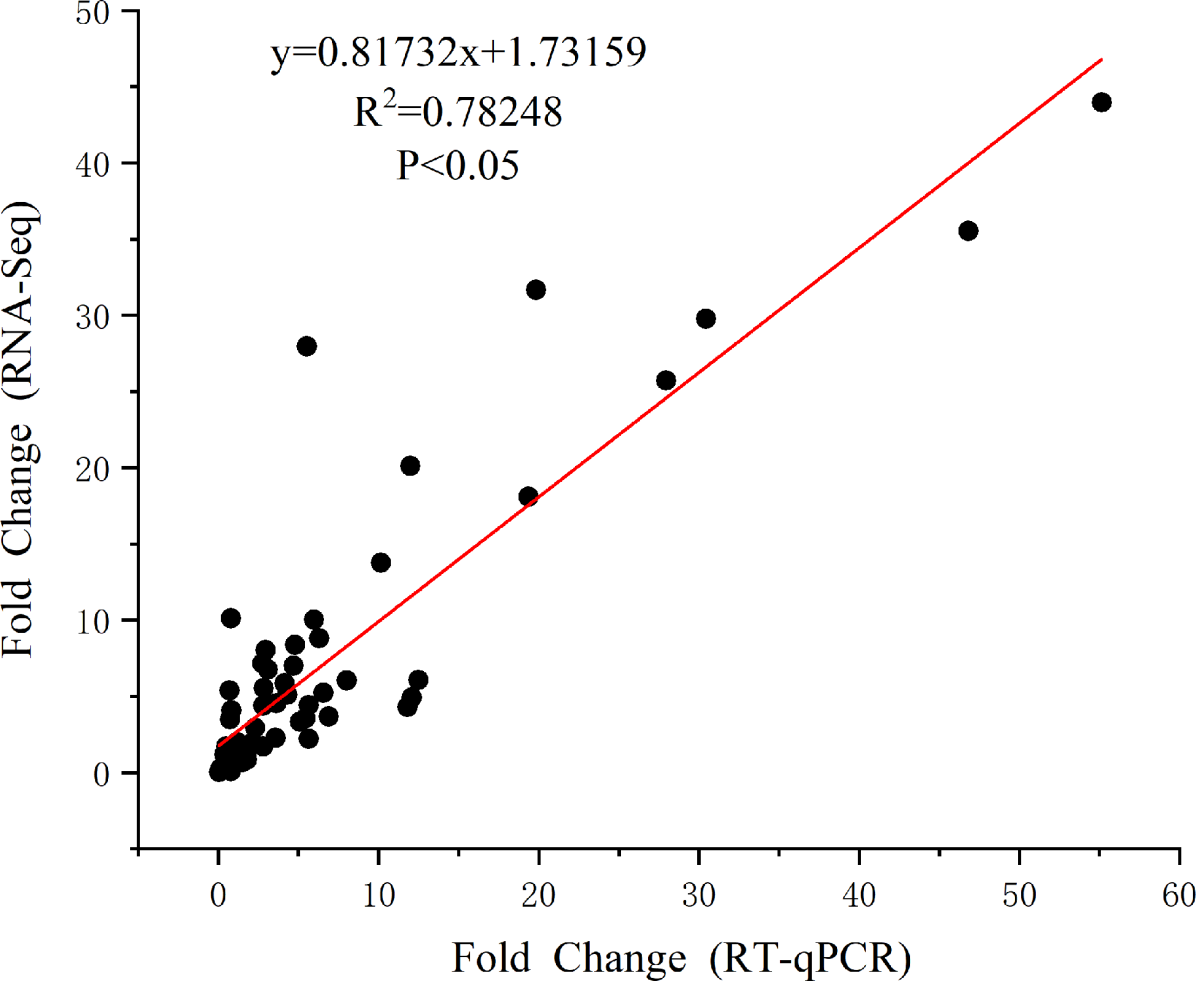
Correlation analysis between RNA-Seq results and RT-qPCR results (p < 0.05).

A total of 20 DEGs that play key roles in flavonoid synthesis and in hormone synthesis and signal transduction, and some DEGs involved in gene and metabolite related networks, were selected for RT-qPCR to verify the accuracy of RNA sequencing. The results showed that the RNA sequencing data correlated significantly with the qRT-PCR results (r^2^ = 0.78259, P < 0.05; **Figures 7**, **8**; **Table S11**), indicating that the results were reliable.

## 4 Discussion

Alfalfa can grow in moderately saline soils; hence, it is cultivated in large areas of coastal China to provide forage for dairy cows. However, alfalfa plants are sensitive to high salt stress, and 50–200 mmol L^-1^ NaCl stress significantly inhibits the growth of alfalfa (Mokhtari et al., 2017). Since alfalfa is cross-pollinated and homologous tetraploid, previous studies on its salt tolerance mechanism have not been carried out in depth. In recent years, with the development of multi-omics and multi-omics data integration analysis technology, transcriptomics, proteomics, and metabolomics analyses have been widely used in the study of alfalfa plant growth and development, and its responses to abiotic stress. This has resulted in new progress in the study of some mechanisms in alfalfa (Song et al., 2016; Chen et al., 2021; Ma et al., 2021; Xu et al., 2021; Yang et al., 2021). In the present study, alfalfa plants were subjected to NaCl stress, root samples were collected for transcriptome sequencing and metabolite detection, and the transcriptome data and metabolome data were integratively analyzed. The transcriptome analysis of alfalfa roots revealed that under NaCl stress DEGs were mainly enriched in isoflavone biosynthesis, flavonoid biosynthesis, ABC transporters, the MAPK signaling pathway, phytohormone signaling, carotenoid biosynthesis, and trans-Zeatin biosynthesis. In addition, metabolomic analysis indicated that under NaCl stress the DAMs in alfalfa were mainly enriched in isoflavone biosynthesis, flavonoid biosynthesis, ABC transporters, alpha-linolenic acid metabolism, and anthocyanin biosynthesis pathways. The integrated analysis of transcriptome and metabolome data showed that isoflavone biosynthesis, flavonoid biosynthesis, and ABC transporter pathways were enriched in alfalfa at 6 and 24 h of NaCl stress. Therefore, based on DEGs and DAMs being enriched in common pathways, this study revealed the molecular mechanism of the rapid response of alfalfa to NaCl stress.

### 4.1 Flavonoid regulatory networks

Flavonoids are common secondary metabolites in plants and can be divided into eight groups according to their molecular structure: flavonols, anthocyanins, proanthocyanidins, isoflavonoids, chalcones, flavones, flavandiols, and aurones (Di Ferdinando et al., 2012). Increased total flavonoid content has been reported in sorghum under moderate and severe salinity stress, with significant changes in related synthetic genes (Ma et al., 2020). The total flavonoid content and the content of seven flavonoids in the leaves of *Cymbopogon* showed an increasing trend with increasing salt concentrations, and short-term salt stress also enhanced antioxidant enzyme activity in the leaves of *Cymbopogon* (Lei et al., 2021). High salt stress significantly increased the expression levels of flavonoid synthesis genes DFR and ANS in rice (Ithal and Reddy, 2004). In this study, the content of some flavonoids such as dihydroquercetin, dihydromyricetin, pelargonidin, luteolin, liquitigenin, and glycitein increased significantly at 24 h of NaCl stress. Moreover, the flavonoid synthesis genes CHS, CHI, FLS, DFR, ANS, F3H, ANR, IFS (2-hydroxyisoflavanone synthase) and F6H (flavonoid 6-hydroxylase) were significantly up-regulated at 1 or 6 h of NaCl stress, which is consistent with the results of Ithal’s study. The CHS gene of okra overexpressed in *Arabidopsis thaliana* increased flavonoid content and the expression levels of SOD and POD genes under salt stress, thereby improving the salt-tolerance of *Arabidopsis* (Wang et al., 2018). Overexpression of the F3H gene increased the content of flavan-3-ols (catechin and epicatechin) and directly improved the antioxidant activity of the tobacco plants (Mahajan and Yadav, 2014). DFR gene overexpression can lead to the accumulation of large amounts of anthocyanins and maintain lower levels of reactive oxygen species and higher chlorophyll content after high salt treatment (Kim et al., 2017). Overexpression of the FLS gene in tobacco increased the total flavonoid content by 1.84-fold compared to that in tobacco in which this gene was not overexpressed. Moreover, it was able to take up more potassium ions to maintain sodium–potassium ion homeostasis and have higher salt tolerance (Wang et al., 2021c). These previous reports indicate that the up-regulation of some important genes in the flavonoid synthesis pathway, alone or in combination, can increase the flavonoid content of plants, thereby enhancing their stress resistance. Additional research has shown that flavonoids can improve plant tolerance to abiotic stresses by directly or indirectly regulating the levels of oxygen-containing radicals, peroxides, and reactive oxygen species, such as oxygen ions (Wang et al., 2021a). In our study, we found that NaCl stress significantly changed the expression levels of CHS, CHI, FLS, DFR, ANS, F3H, and IFS in the flavonoid synthesis pathway of alfalfa. Moreover, the concentrations of some flavonoid products such as licorice, dihydroquercetin, and luteolin increased significantly under NaCl stress for 24 h. The transcriptome GO was also enriched in some functional units such as redox-regulation process and flavonoid synthesis of secondary metabolites, while the transcriptome and metabolome KEGG were enriched in common pathways such as isoflavone biosynthesis, flavonoid biosynthesis, and ABC transport. The ABCC2 transporter protein can co-transport flavonoid compounds such as anthocyanin (Cy3G) with glutathione (Behrens et al., 2019). Moreover, the ABCG10 transporter protein can transport the isoflavone synthesis precursors, coumarate 4-coumarate, and liquiritigenin (Biala et al., 2017). The results of this study indicated that the regulation of flavonoid synthesis and transport is an important mechanism for alfalfa to adapt to salt stress.

### 4.2 Plant hormone regulatory networks

Phytohormones are trace endogenous signaling molecules synthesized by plants, which play an important role in stress response. Under salt stress, GA content decreases and the expression of GA20ox, a key gene for GA synthesis, is down-regulated (Zhu et al., 2019). Salt stress inhibits plant growth by reducing GA levels, whereas exogenous application of GA stimulates the synthesis of endogenous GA in plants, thus promoting plant growth (Niharika et al., 2020). The repressor DELLA protein can inhibit the expression of transcription factor PIF3/4 in the gibberellin transduction pathway, and the amycin receptor protein genes 1 and 2 (GID1 and GID2, respectively) can attenuate DELLA protein after binding to GA. In the present study, the GA20ox in alfalfa roots was down-regulated at 6 and 24 h of NaCl stress, while the contents of GA1, GA4, and GA9 were decreased at 1 and 6 h of NaCl stress and significantly increased at 24 h of NaCl stress. The genes of DELLA, GID1, and PIF3/4 in the gibberellin transduction pathway of alfalfa roots were significantly down-regulated at 6 h, but most of them were restored to pre-stress levels, or even up-regulated, at 24 h. These results indicated that alfalfa plants can adapt to a salt stress environment by reducing GA content to inhibit plant growth in the early stage of NaCl stress. With the duration of salt stress, alfalfa plants gradually adapted to its effects, and the GA content was increased again to restore plant growth.

ABA is a hemiterpene phytohormone, and the addition of 10 µM ABA to the root zone of wheat under salt stress significantly increases aboveground biomass and root length, reduces sodium uptake by more than 80%, and increases antioxidant enzyme activity in roots (Parveen et al., 2021). Endogenous ABA can improve salt tolerance of rice by regulating stomatal closure and improving water balance (Shahzad et al., 2017). In this study, the ABA content in roots did not change significantly after NaCl stress, while ABA synthesis-related genes were up-regulated. A possible explanation of this is that ABA synthesized by roots is transported to the ground and is involved in the regulation of shoot defense against salt stress (Poór et al., 2019). The SnRK2 kinase can induce the expression of transcription factor ABF2 in the ABA transduction pathway of alfalfa. Protein phosphatase PP2C inhibits SnRK2 kinase activity by dephosphorylation, while receptor protein PYL inhibits PP2C activity by ABA. In a previous study, overexpression of ABF2, a transcription factor for ABA synthesis in *Arabidopsis*, was shown to increase the expression of the stress response gene KIN2, thereby improving *Arabidopsis* salt tolerance (Zhao et al., 2016). In the present study, while PYL and SnRK2 genes were down-regulated under 6 and 24 h of NaCl stress, ABF2 and PP2C gene expression increased, suggesting that alfalfa can enhance the expression of downstream resistance genes in roots by increasing the expression of the transcription factor ABF2, thereby enhancing the resistance of the plants to salt stress.

JA is an important stress regulatory hormone in plants. MeJA, and JA isoleucine (JA-ile) are JA analogs (Shimizu et al., 2010; Wang et al., 2021d). Transcriptome analysis of sweet potato roots under salt stress revealed significant up-regulation of genes related to JA synthesis and signal transduction in salt-tolerant lines compared with that in salt-sensitive lines (Zhang et al., 2017). The JA and JA-ile in *Medicago truncatula* increased by 3 and 6 times, respectively, after 1 h salt stress (Domenico et al., 2019). In the present study, the genes related to JA synthesis (LOX, AOS, AOC, and OPR3) were significantly up-regulated in alfalfa roots after 1 or 6 h NaCl treatment, and meJA content increased significantly at 24 h of NaCl treatment. These results indicated that salt stress can lead to an increase in JA content in plants (Prerostova et al., 2017). It has been shown that foliar spraying of JA on soybean under salt stress reduces sodium uptake while increasing betaine content, membrane stability index, and leaf water content (Farhangi-Abriz and Ghassemi-Golezani, 2018). It has been reported that JA acts through and is central to a regulatory network of multiple hormone signals (Yang et al., 2019). Moreover, MeJA can increase GA3 levels in plants (Gao et al., 2021), and significantly increase the expression of ABF2, ABF8, and ABF10 under 24 h of NaCl stress (Lu et al., 2021). In this study, MeJA content started to rise after 1 h of NaCl stress and increased significantly at 24 h of NaCl stress, GA content increased significantly at 24 h of NaCl stress, and ABF2 was significantly up-regulated under NaCl stress for 1 h. These results indicated that GA, ABA, and JA were all involved in the defense response of alfalfa under NaCl stress to some extent, and JA was possibly a key link in this hormonal regulatory network.

### 4.3 Association between flavonoids and phytohormones

Overexpression of genes in the GA and JA synthesis pathways and the ABA and cytokinesis (CK) transduction pathways can change flavonoid content in plants, suggesting that flavonoid content changes may be achieved through hormonal regulation of the competition between FLS and DFR (Wan et al., 2020). Treatment of soybean with exogenous GA3 under NaCl stress has been similarly shown to increase GAs (GA1 and GA4) and JA, as well as isoflavone (daidzein and genistein) content, and reduce salt stress injury (Hamayun et al., 2010). The ABCC2 transporter protein can transport ABA, which is involved in the transport of flavonoid compounds (Behrens et al., 2019). In this study, the contents of GAs, MeJA, and isoflavones and some flavonoid compounds in alfalfa roots significantly increased at 24 h of NaCl stress, and DEGs and DAMs were also enriched in the ABC transporter pathway after 1 h of NaCl stress. The results also showed that dihydroquercetin and LUT5 of the flavonoid synthesis pathway were significantly correlated with ABF2, PP2C, and PYL genes of the hormone synthesis pathway in alfalfa roots, suggesting that GA and ABA were involved in flavonoid synthesis by regulating the expression of ABF2, PP2C, and PYL to enhance the salt resistance of alfalfa plants. Transcriptomic and metabolomic analyses of alfalfa roots revealed that 250 mM NaCl treatment resulted in alterations in several pathways, including phenylalanine metabolism, flavonoid synthesis, and α-linolenic acid metabolism, with significant up-regulation of the AOS, AOC, LOX, and OPR genes in the JA synthesis pathway. In addition, JA acts as a signaling molecule to promote flavonoid biosynthesis and induce flavonoid accumulation to adapt to salt stress (Zhang et al., 2021b). MeJA increases the isoflavone content and antioxidant activity of *Astragalus trichocarpa* roots and induces up-regulation of the CHI and CHS genes (Gai et al., 2016). MeJA treatment at 25–50 µM significantly induced anthocyanin accumulation in red fenugreek roots (Yasuhiro et al., 2010). In this study, the significant up-regulation of genes related to JA synthesis at 1 or 6 h of salt stress was the same as the expression pattern of genes related to flavonoid synthesis, and the concentration of flavonoids was significantly increased at 24 h of stress. (+)-7-Jasmonate isomethyl ester was significantly correlated with FLS (MsG0480022886.01), which is consistent with the above results.

## 5 Conclusion

We used transcriptomic and metabolomic approaches to study the changes in gene transcript levels and metabolite content in alfalfa plants under NaCl stress. Through the association analysis of metabolic pathways, it was found that flavonoids and phytohormones interact to jointly participate in the adaptation of plants to salt stress. The results showed that most of the flavonoid synthesis genes, as well as JA synthesis and transduction genes, were significantly up-regulated at 1 or 6 h of NaCl stress. The expression of most genes in the GA and ABA synthesis and transduction pathways also changed significantly during NaCl stress. Moreover, the levels of six flavonoids (dihydroquercetin, dihydromyricetin, anemone, lignan, glycyrrhizin, and glycine), three GA products (GA1, GA4, and GA9), and two jasmonate products ((+)-7-isomethyl jasmonate and methyl jasmonate) increased significantly at 24 h of NaCl stress. A combined analysis of DEGs and DAMs revealed that LUT5 (MsG0880047112.01) and three ABA transduction genes (MsG0180000344.01, MsG0880047375.01, MsG0380015520.01) were significantly correlated with dihydroquercetin. The flavonoid Luteolinidin was significantly correlated with PIF4 (MsG0180003748.01) and two PP2C (MsG0180000980.01, MsG0480023807.01). (+) -7-isomethyl jasmonate was significantly correlated with the FLS (MsG0480022886.01) gene. In summary, flavonoids, GA, JA and ABA all play important roles in alfalfa’s response to salt stress. The flavonoid products dihydroquercetin and luteolin were significantly correlated with several ABA and GA genes, and the JA product (+) -7-isomethyl jasmonate was significantly correlated with FLS genes. In addition, (+) -7-isomethyl jasmonate and homoeriodictyol chalcone were significantly correlated with POD (MsG0280008067.01). POD was also significantly up-regulated under NaCl stress for 6 and 24 h. These results indicate that GA, ABA, and JA may be involved in the regulation of flavonoids to improve the salt tolerance of alfalfa. JA and flavonoid-related genes and metabolites have similar expression patterns under NaCl stress, which may be the key signal to promote the synthesis of flavonoids. In conclusion, these results reveal the possible molecular mechanism of alfalfa’s adaptation to salt stress, identify a number of candidate genes, and provide new insights for the study salt tolerance in alfalfa. Future studies will focus on which genes are involved, and how JA links flavonoids and various plant hormones to participate in the salt tolerance of alfalfa.

## Data availability statement

The original contributions presented in the study are publicly available. This data can be found here: NCBI, PRJNA892760.

## Author contributions

XW conceived and designed the research. JY, JW, and JL performed the experiments. JY analyzed the data, and wrote the paper. XW put forward valuable suggestions on the modifications of the paper. All authors contributed to the article and approved the submitted version.

## Glossary

**Table:**

ABA	abscisic acid
ABF2	ABA responsive element binding factor 2
ANS	anthocyanin synthase
AOC	allene oxide cyclase
AOS	allene oxide synthase
CAT	catalase
CHI	chalcone isomerase
CHS	chalcone synthase
DAM	differential accumulation metabolite
DEG	differentially expressed gene
DFR	dihydroflavanol reductase
ERF1	ethylene-responsive transcription factor 1
F3H	flavanone hydroxylase gene
FLS	flavonol synthase
GA	gibberellin
GA20ox	gibberellin 20 oxidase
GA3ox	gibberellin 3beta-dioxygenase
GO	Gene Ontology
IFS	2-hydroxyisoflavanone synthase
JA	jasmonic acid
JA-ile	jasmonic acid isoleucine coupling
KEGG	Kyoto Encyclopedia of Genes and Genomes
KIN2	stress-induced protein kinase 2
LOX	13-lipoxygenase
LUT5	beta-ring hydroxylase
MDA	malonaldehyde
MeJA	jasmonic acid methyl ester
miaA	tRNA dimethylallyl transferase
NCED	9-cis-epoxycarotenoid dioxygenase
OPR3	12-oxophytodienoic acid reductase
PCA	principal component analysis
PIF3/4	phytochrome-interacting factor 3/4
PLS-DA	partial least-squares discriminant analysis
POD	peroxidase
PP2C	protein phosphatase PP2C
PR1	pathogenesis-related protein 1
PRO	proline
PYL	ABA receptor PYL2
QC	quality control
RR	response regulator
SNRK2	serine/threonine-protein kinase SRK2
SOD	superoxide dismutase
VIP	variable important for the projection
VSP2	vegetative storage protein 2
