# Supplementary material for: Integrative analysis of transcriptome and metabolome revealed the mechanisms by which flavonoids and phytohormones regulated the adaptation of alfalfa roots to NaCl stress
Source: Front Plant Sci. 2023 Feb 3;14:1117868. doi: 10.3389/fpls.2023.1117868 (PMC9936617; doi:10.3389/fpls.2023.1117868)
Supplement: Supplementary file 1 [file DataSheet_1.zip › Supplementary_Material.docx]

Supplementary Material

# Supplementary Figures and Tables

## Supplementary Figures

**Supplementary Figure S1**. Analysis of NaCl stress at 1h, 6h, 24h and common 756DEGs bubble map (Q-value < 0.05).

**Supplementary Figure S2**. Volcanic map analysis of 1, 6 and 24hDAMs under NaCl stress (VIP ≥ 1; fold change ≥ 1.2 or ≤ 0.8333; Q-value < 0.05).

**Supplementary Figure S3**. Changes of six biochemical indexes in alfalfa roots under NaCl stress. A. POD activity. B. SOD activity. C. CAT activity. D. MDA content. E. BCA protein content. F. PRO content. * and ** represent P <0.05 and P <0.01 respectively.

**1.2 Supplementary Tables.**

**Supplementary Table S1.** Transcriptome Sequencing results.FPKM

**Supplementary Table S2.** PLS-DA model parameter.

R2: Represents the explanatory ability of the model. The closer R2 value is to 1, the better the explanatory ability of the PLS-DA model. Q2: Represents the prediction ability of the model. The closer Q2 value is to 1, the better the prediction ability of the PLSDA model.

**Supplementary Table S3.** Differential metabolite test results

**Supplementary Table S4.** Annotation information of differential metabolites

**Supplementary Table S5.** Changes of six biochemical indexes in alfalfa roots under NaCl stress.

**Supplementary Table S6.** Expression of genes related to flavonoid biosynthesis.

**Supplementary Table S7.** Expression of metabolites related to flavonoid biosynthesis

**Supplementary Table S8.** Expression of genes involved in the synthesis and transactivation of phytohormone..

**Supplementary TableS9.** Expression of relevant metabolites in the synthesis and transactivation of phytohormone.

**Supplementary Table S10.** Gene and metabolite related networks

**Supplementary Table S11.** RT-qPCR primer sequence..
