# Supplementary figures and images for: Integrative analysis of transcriptome and metabolome revealed the mechanisms by which flavonoids and phytohormones regulated the adaptation of alfalfa roots to NaCl stress
Source: Front Plant Sci. 2023 Feb 3;14:1117868. doi: 10.3389/fpls.2023.1117868 (PMC9936617; doi:10.3389/fpls.2023.1117868)

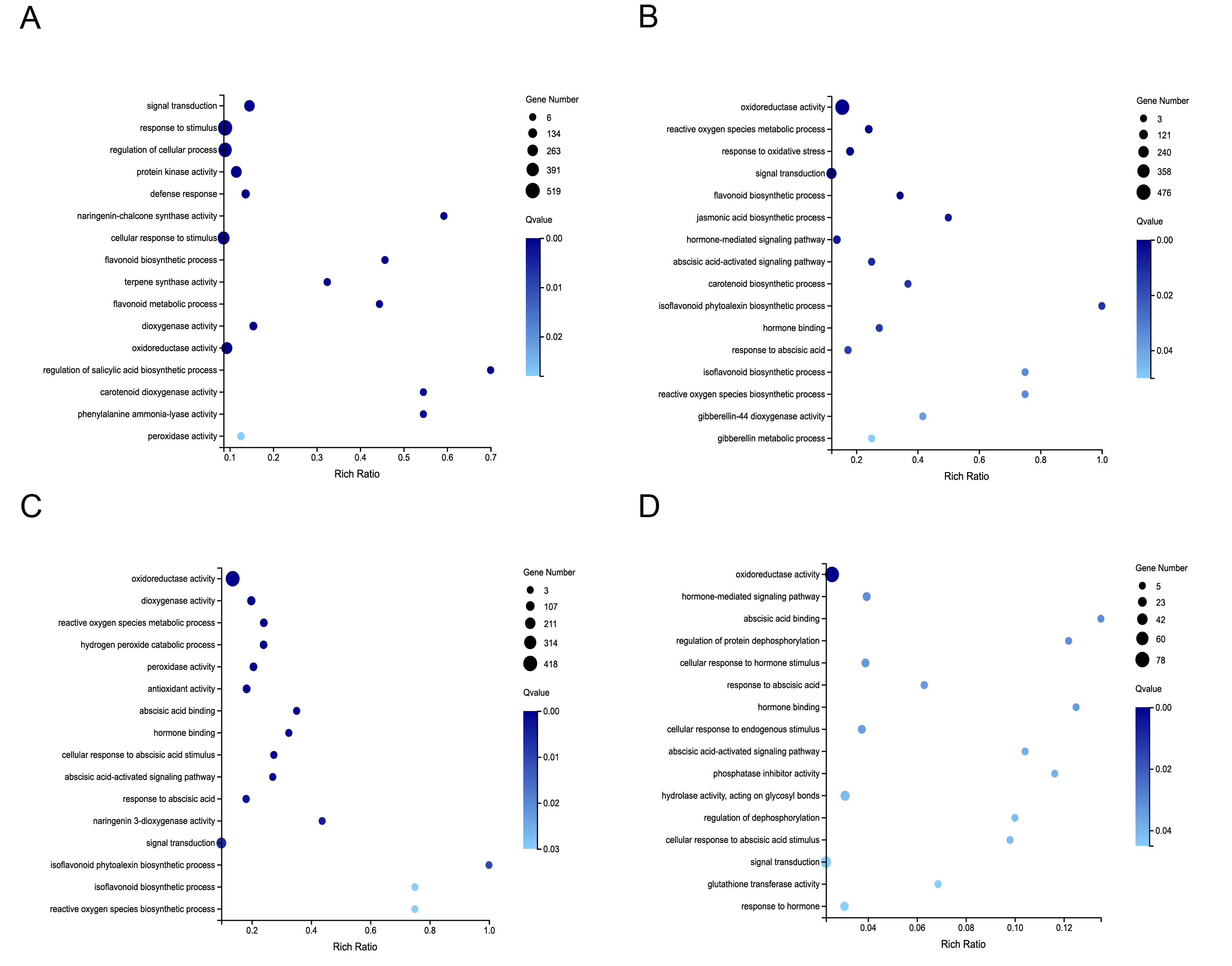

Supplement: Supplementary file 1 [file DataSheet_1.zip › Figures/FigureS1.tif]

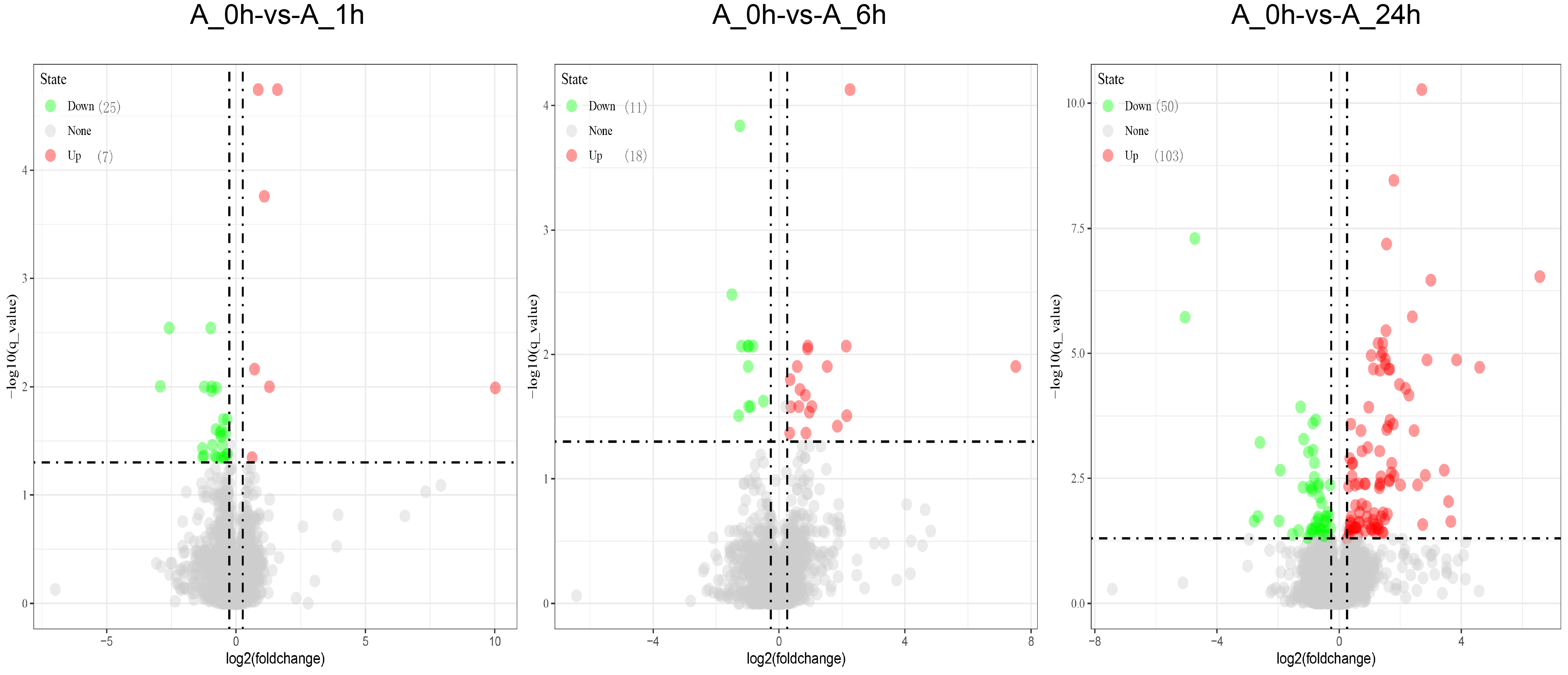

Supplement: Supplementary file 1 [file DataSheet_1.zip › Figures/FigureS2.tif]

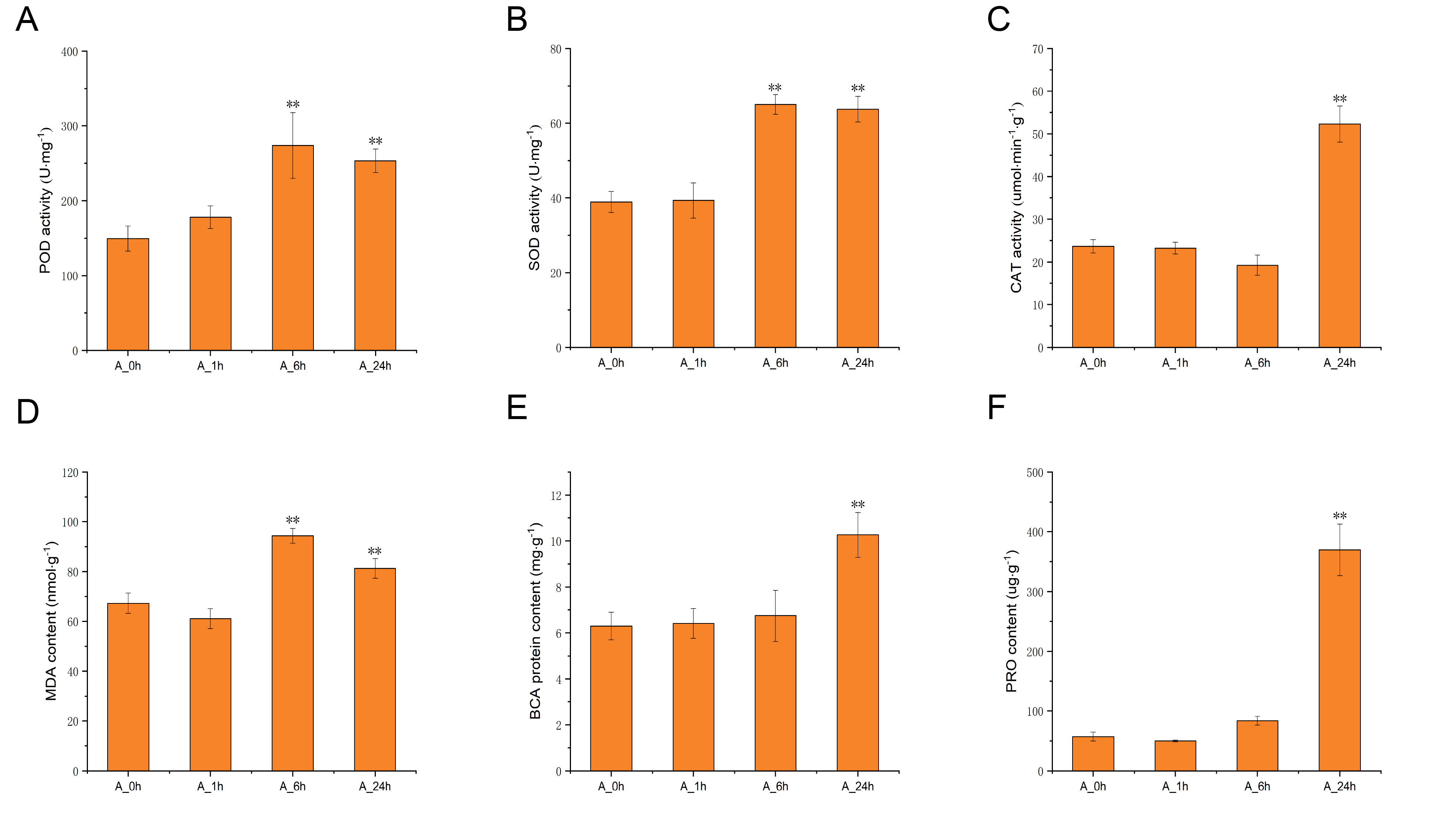

Supplement: Supplementary file 1 [file DataSheet_1.zip › Figures/FigureS3.tif]
